# The Efficacy of Organo-Complex-Based Wood Preservative Formula Against Dry-Wood Termite *Cryptotermes cynocephalus* Light

**DOI:** 10.3390/insects2040491

**Published:** 2011-11-15

**Authors:** Maya Ismayati, Khoirul Himmi Setiawan, Didi Tarmadi, Deni Zulfiana, Sulaeman Yusuf, Budi Santoso

**Affiliations:** 1 Research and Development Unit for Biomaterials, Indonesian Institute of Sciences (LIPI), Bogor 16911, Indonesia; E-Mails: khoirul_himmi@biomaterial.lipi.go.id (K.H.S.);didi@biomaterial.lipi.go.id (D.T.); zulfiana@yahoo.com (D.Z.);sulaeman@biomaterial.lipi.go.id (S.Y.); 2 PT. Karuna Sumber Jaya, Cileungsi, Bogor 16820, Indonesia; E-Mail: info@palletindonesia.co.id

**Keywords:** Organo-Complex formula, dry-wood termite, *Cryptotermes cynocephalus* Light

## Abstract

The utilization of pesticides often leaves residues which potentially pollute the environment. This journal issue has been encouraging some researchers to find an environmentally friendly insecticide by a cheaper wood preservative method. The International Standard for Phytosanitary Measures 15 (ISPM 15) [1] that is adopted in wood packaging protection in Europe is not suitable for tropical countries like Indonesia. Therefore, the treatment by Organo-Complex-based wood preservation, which consists of copper chromium combined with natural organic compounds, is proposed for effective treatment at a lower cost. The bioassay test was subjected to dry wood termite *Cryptotermes cynocephalus* Light. The result showed that wood materials treated by 10 ppm Organo-Complex formula gave good results which were indicated by the low consumption and the fast termination of the termites. The toxicity analysis of C-C organic compound solution is classified as grade IV (WHO, 2003) [2], or not harmful. Analysis of the residual content four weeks after the spraying treatment showed a significant reduction in the inorganic content (copper chromate complex), in the range of 35%, and in extracts of natural materials (natural extracts), above 80%.

## 1. Introduction

Methods of wood packaging product protection differ in each country. As a tropical country, Indonesia has higher humidity than European countries; therefore, the wood packaging protection in Indonesia differs from those countries with a less humid climate. In addition, there are differences in the raw materials used in wood packaging products. In Indonesia, wood packaging manufacture is a home industry using wood from local suppliers resulting in packing material composed of various kinds of wood species and unknown age. Furthermore, the International Standard for Phytosanitary Measures 15 (ISPM 15) [1] adopted in wood packaging protection in Europe is not suitable for Indonesia. Thus, it is necessary to find a new preservative method of wood packaging product in accordance with the climate conditions in Indonesia and with a relatively cheaper cost.

Environmental pollution issues have encouraged some researchers to find environmentally friendly insecticides. Insecticides are generally tested for oral and dermal toxicity. The content analysis for residuals provides information on how much material is left on wood packaging products after treatment. Residual contents should not exceed the required standard for the country where the packing material is used or shipped. The aim of this research was to evaluate the efficacy of an Organo-Complex-Based Wood Preservatives Formula against the dry-wood termite *Cryptotermes cynocephalus* Light and to study residual contains on the wood products treated as well as its toxicity to mice.

## 2. Experimental Section

### 2.1. Dry-Wood Termite Test

**Figure 1:**
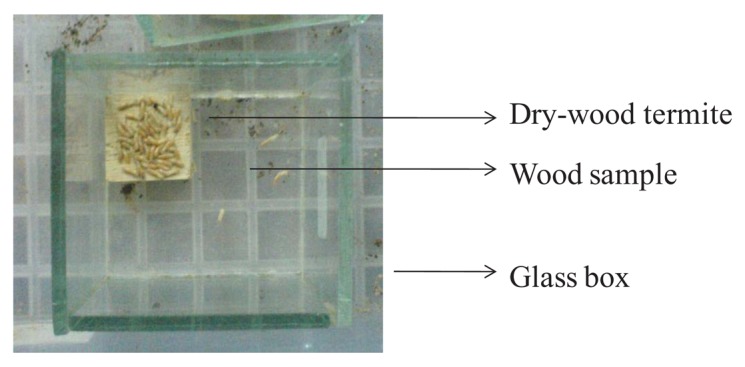
Glass box of dry-wood termite test [3].

The chemical solution used in this study consisted of a combination of sodium bichromate, copper (II) sulphate, *Niccotiana tobaccum* and *Eugenia carryophillata* extracts. Test blocks were exposed to a dry wood termite forced-feeding test according to Indonesian National Standard (SNI) 01-7207-2006 [3] Wood samples (2 cm × 2 cm × 1 cm) treated with the chemical substance solution at 10 ppm CC-organic compound (1:1), were placed in glass box size 4 × 4 × 4-cm with 50 workers of dry-wood termite (Figure 1) and the experiment was replicated 3 times. Termite mortality was observed once per week for 6 weeks and termite mortality and weight loss of wood sample (rubber wood) was determined at the end of observation.

Wood samples were characterized for durability by the test scale in Table 1 using average values obtained from the weight loss value of each replicate.

### 2.2. Toxicity Test of Organo-Complex-Based Wood Preservatives Formula

The toxicity test consisted of oral and dermal toxicity according to Directorate of Fertilizers and Pesticides [4]. It was conducted in the Faculty of Veterinary Medicine, Bogor Agricultural University, Indonesia.

### 2.3. Residual Chemical Tests of Wood Treated by Organo-Complex-Based Wood Preservatives Formula

#### 2.3.1. Analysis of Metal Content (Cu and Cr)

Cu and Cr metal content was analyzed by the atomic absorption spectroscopy analysis method (Atomic absorption spectroscopy, AAS) using Perkin Elmer Zeeman AAS instrument 5100. The preparation wet destruction was using *Aqua regia*, HNO3:H2SO4 = 1:1. The specimen wood used was sengon (*Enterolobium cyclocarpum*) size of 2 cm × 2 cm × 1 cm. Analysis of inorganic materials content (copper chromate complex) was conducted using AAS with preparations of test specimens of the treated wood, and wood specimens of the control. Analysis was conducted with flame AAS (flame) using a standard curve for Cu and Cr generated using concentrations of 1.25, 2.5, 5.0, 7.5 and 10.0 ppm.

#### 2.3.2. Analysis of Organic Content

The testing of organic content and natural ingredients extractive was performed by High Performance Liquid Chromatography (HPLC) using Shimadzu HPLC instrument [5]. The test specimens were prepared by the soxhletation method using methanol solvent.

## 3. Results and Discussion

**Figure 2:**
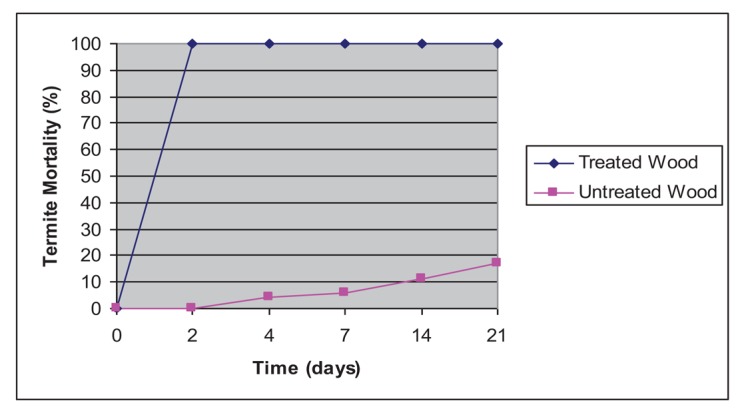
Termite mortality rate of essential oil evaluated.

The results of the bioassay test of C-C organic compound solution are presented in Table 2 and Figure 2. In this study, rubber wood (*Hevea brasiliensis*) with less durability was used as control. There were significant differences in weight loss percentage between the untreated wood and the control. The average value of untreated wood weight loss was 8.6%, showing low resistance against dry wood termite. Meanwhile, wood treated with the C-C organic compound solution showed high resistance against dry wood termite with an average value of 1.4%. It can be concluded that C-C organic compound solution can be classified as Grade I or high resistant against the dry-wood termite *C. cynocephalus* Light.

Termiticide activity of the C-C organic compound solution was indicated by the termite mortality data. Figure 2 shows the average value of termite mortality on treated and untreated wood. The rubber wood control showed 17% termite mortality at day 21 while, by the second day of testing, all treatments resulted in 100% mortality.

Our results support previous studies that show that C-C organic compound solution has insecticide activity towards *Coptotermes gestroi* and Powder Post Beetle *Heterobostrychus aequalis* [6,7]. Damage caused by dry wood termite attack is difficult to detect as these termites usually leave a thin layer under the surface of the attacked wood and they do not require high levels of moisture like subterranean termites. The mortality table above shows that the termites died in only 2 days. It is very important for the preservation of wood packaging if there is a dry wood termite attack that they can quickly be exterminated. Finally, this preservation method can reduce consumer claims resulting from unwanted infestations. However, further analysis is necessary, particularly for toxicity and residual analysis after field applications.

Oral and dermal toxicity testing of C-C organic compound solution was conducted. In the oral toxicity test, five male mice were treated with a single dose (5,000 mg/kg body weight) and changes in behavior and physiological reactions observed at 1 hour, 2 hours, 3 hours, 4 hours and 24 hours after treatment. Each dose was replicated 3 times. Observations in this test consisted of body weight, mortality and clinical symptoms until the fourteenth day of testing. Results showed that there was no mortality of mice after treatment with C-C organic compound solution but there were changes in mice behavior exhibited by symptoms of excitation, weakness and hair loss. During 14 days of observation, mice treated with the C-C organic compound solution showed weight loss until the sixth day that continued until the end of the test periods. The results of mouse body weight at 14 days are shown in Table 3.

**Figure 3:**
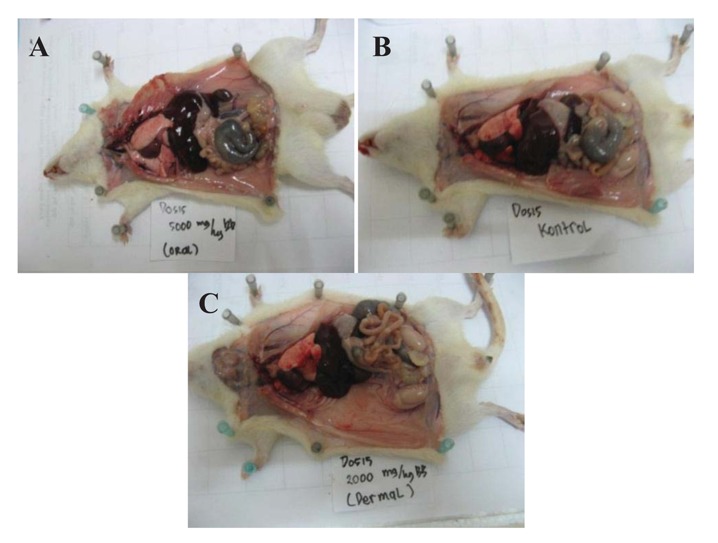
Pictures of the histological evidence of the visceral organ of the mice, control (**A**), oral treatment (**B**) and dermal treatment (**C**).

Dermal toxicity was tested on mice treated at the dose of 2,000 mg/kg body weight. Within 14 days of observation, there was no mouse mortality or hair loss. Otherwise, there was no abnormal behavior. The skin of mice was normal throughout the test period and we did not observe any abnormal behavior such as skin irritation, redness or other hypersensitivity symptoms. During test observation, the body weight of mice treated by C-C organic compound solution still increased, as reported in Table 4. The macroscopic observations (Figure 3) on visceral organ groups of mice treated with C-C organic compound solution showed changes in anatomical pathology such as congestion in lungs and liver. However, generally there was no damage to organ or tissue, as determined by macroscopic observation, of the visceral organs.

The macroscopic observations on visceral organ groups on mice given oral treatment with C-C organic compound solution showed changes in anatomical pathology such as dilatation on the right and left ventricular hypertrophy in the heart, pneumonia and congestion of the lungs, congestion in the kidneys, liver and lymph and degeneration of the liver. Analysis of the residual content was conducted to determine the extent of pesticide content of the material left in the wood product packaging treated by a C-C organic compound solution. The results of the inorganic contents of the packaging material are shown in Table 5.

Based on Table 5, concentrations of Cu and Cr from the spray treatment on wood were 2.40 mg/L and 2.06 mg/L, respectively. The concentration of Cr (2.06 mg/L) is higher than maximum contamination level (MCL) value issued by the US EPA (Environmental Protection Agency) at 1.0 mg/L. However, this dose is still permitted in Indonesia where the recommended concentration of wood preservative agent for wood protection treatment is about 5 kg/m3. In addition, the process of spraying CC-Organic solution is ruled by Standard Operating Procedure (SOP) with the intent of reducing chemical contamination. This means that the spraying process has been conducted under proper conditions using the proper safety tools. Analysis of organic content results using high performance liquid chromatography (HPLC) shows that natural extractive materials are presented as shown in Table 6. The preparation of test specimens was carried out with the soxhletation method using methanol as solvent.

Table 6 shows that the extract content of tobacco has the highest residues as indicated by the peak intensity (peak retention 3,640). The results of the chromatographic analysis (HPLC) showed a 80% decrease in the content natural extractive materials from wood packaging products by the fourth week. In fact, acetic acid decreased by 100% by the fourth week after spraying the C-C organic compound solution. This can be explained by the fact that natural ingredients such as citrus and cloves are volatile compounds.

## 4. Conclusions

The present study has confirmed that CC-Organic compound solution has a strong termicidal activity against dry wood termite *Cryptotermes cynocephalus* Light and is classified as grade IV [2], or not harmful. Residual content analysis in the fourth week after spraying treatment showed a significant reduction in both the inorganic content (copper chromate complex), nearly 35%, and of natural materials extracts, above 80%.

## Figures and Tables

**Table 1:** Durability of wood sample based on weight loss [3].

**Grade**	**Durability**	**Weight Loss (%)**
I	Highly Resistant	<2.0
II	Resistant	2.0–4.4
III	Moderate	4.4–8.2
IV	Non-Durable	8.2–28.1
V	Susceptible	>28.1

**Table 2:** The result of weight loss percentage of dry wood termite test.

**Wood Specimens**	**Average Value (%)**	**Grade Level**
Untreated wood	8.6	IV (Non-Durable)
Wood treated by CC-organic compound (1:1)	1.4	I (High Resistant)

**Table 3:** Average body weight groups of Sprague Dawley mice receiving treatment of acute oral toxicity, after treated by C-C organic compound solution with a dose of 5,000 mg/kg.

**Test Periode (day)**	**Body Weight (BW)of Mice (g)**	

	**Untreated Mice**	**Treated Mice(5,000 mg/kg BW)**
0	208.84	221.28
1	210.51	202.32
2	211.28	205.28
3	213.49	208.11
4	215.85	209.94
5	216.71	210.51
6	218.49	212.30
7	220.06	213.63
8	222.77	215.58
9	224.51	217.61
10	226.26	219.29
11	227.48	221.84
12	229.95	223.53
13	231.61	224.47
14	234.25	226.59

**Table 4:** Average weight loss group Sprague Dawley mice receiving treatment of acute dermal toxicity, after treated by C-C organic compound solution with a dose 2,000 mg/kg.

**Test Periode (day)**	**Body Weight (BW) of Mice (g)**	

	**Untreated Mice**	**Treated Mice (2,000 mg/kg BW)**
0	208.84	206.94
1	210.51	207.51
2	211.28	209.29
3	213.49	211.50
4	215.85	212.73
5	216.71	214.48
6	218.49	215.59
7	220.06	216.71
8	222.77	218.29
9	224.51	221.07
10	226.26	223.84
11	227.48	225.57
12	229.95	228.31
13	231.61	230.22
14	234.25	231.48

**Table 5:** Inorganic compound (copper chromium complex) of treated wood specimens.

**Sample Specimens**	**Cu (mg/L)**	**Cr (mg/L)**
Control (Untreated wood)	0.075	0.020
CC-Organic compound solution	4,603	3,963
Wood treated by CC-Organic compound solution	2.04	2.06

**Table 6:** Percentage of organic content on wood samples.

**Sample Specimens**	**Initial Intensity**	**Residual Intensity (after 4 weeks)**	**Decreasing Percentage**
Acetic acid	9,186.81	-	100%
Tobacco extract	29,464.18	4,467.97	84.84%
Citric acid	17,315.82	545.39	96.85%
Clove extract	7,832.53	54.42	99.30%
